# A cross-sectional study to estimate the point prevalence of painful diabetic neuropathy in Eastern Libya

**DOI:** 10.1186/s12889-018-6374-9

**Published:** 2019-01-17

**Authors:** Sabri Garoushi, Mark I. Johnson, Osama A. Tashani

**Affiliations:** 1 Centre for Pain Research, School of Clinical and Applied Sciences, Portland Way, Leeds Beckett University, Leeds, LS1 3HE UK; 2 MENA Research Group, School of Clinical and Applied Sciences, Leeds Beckett University, Leeds, UK; 3 Faculty of Medicine, University of Benghazi, Benghazi, Libya

## Abstract

**Background:**

Painful Diabetic Neuropathy (PDN) is a complication that affects up to one third of people living with diabetes. There is limited data on the prevalence of PDN from countries in the Middle East and North Africa. The aim of this study was to estimate the point prevalence of PDN in adults in Eastern Libya using the self-report Leeds Assessment of Neuropathic Symptoms and Signs (S-LANSS) pain scale.

**Methods:**

We invited patients attending the Benghazi Diabetes Centre who had diabetes for ≥ 5 years to take part in the study. Patients provided consent and completed the Arabic S-LANSS. Anthropometrics, marital status, socioeconomic and education information was recoded and fasting plasma glucose concentration determined.

**Results:**

Four hundred and fifty participants completed the study (age = 19 to 87 years, BMI = 17.6 to 44.2 kg/m^2^, 224 women). One hundred and ninety five participants (43.3%) reported pain in their lower limbs in the previous 6 months and 190/195 participants (97.4%) reported a S-LANSS score of ≥ 12 suggesting they had neuropathic pain characteristics. Thus, 42.2% (190/450) of participants with diabetes were categorised as experiencing pain with neuropathic characteristics. Mean ± SD duration of diabetes for participants with PDN (20.4 ± 6.5 years) was significantly higher compared with those without PDN (11.1 ± 4.6 years). Participants with PDN smoked tobacco for more years than those without pain (7.9 ± 12.3 years versus 1.1 ± 3.9 years respectively); had significantly higher fasting plasma glucose concentration (143.6 ± 29.3 mg/dl versus 120.0 ± 17.3 mg/dl) and had a significantly higher levels of education and employment status. The most significant predictors of PDN were duration of diabetes (OR = 25.85, 95% CI = 13.56–49.31), followed by smoking for men (OR = 8.28, 95% CI = 3.53–9.42), obesity (OR = 3.96, 95% CI = 2.25–6.96) and high fasting plasma glucose concentration (OR = 3.51, 95% CI = 1.99–6.21).

**Conclusion:**

The prevalence of PDN in people with diabetes in Eastern Libya was 42.2%. Risk factors for developing PDN were high fasting plasma glucose concentration, long duration of diabetes, and higher level of educational and employment status.

**Electronic supplementary material:**

The online version of this article (10.1186/s12889-018-6374-9) contains supplementary material, which is available to authorized users.

## Background

Diabetes is a global health care problem and financially costly. The cost of treating complications arising from diabetes is 3 to 4 times more than the cost treating diabetes. Data about the financial cost and burden of diabetes on resource-limited countries is lacking, with few estimates of prevalence of the disease or its complications. It has been estimated that the point prevalence of diabetes in the Middle East and North Africa (MENA) in 2017 was 38.7 million (11% of the population) [1, 2]. In 2017, the International Diabetes Federation (IDF) estimated that the total financial health care costs of treating diabetes and its complications in the MENA region was equivalent to £15.45 billion GBP ($20.5 billion USD). This equates to 15% of total health costs and higher than the World as a whole (11.3%), Europe (10%) and Africa (5.7%) [2]. Projections suggest that the incidence of diabetes in the MENA region will double by 2045 and financial costs will rise to £28 billion GBP ($37 billion USD) by 2045 [2].

Painful Diabetic Neuropathy (PDN) is a common complication of diabetes affecting up to one third of patients worldwide. Diagnosis, treatment and management of PDN can be challenging for clinicians because of the complexity of pathophysiological mechanisms contributing to pain [3, 4]. Tools that capture a person’s self-report of their pain experience are available to assist clinicians screen for the presence of probable neuropathic pain [5]. Examples include: The Leeds Assessment of Neuropathic Symptoms and Signs (LANSS) [6]; the Neuropathic Pain Questionnaire (NPQ) [7]; Douleur Neuropathique en 4 questions (DN4) [8]; painDETECT [9]; ID-Pain [10] and the Standardized Evaluation of Pain (StEP) [11]. Management of PDN in the MENA region appears to be inadequate with limited use of neuropathic screening tools and a paucity of data on the extent of the problem, especially in the Maghreb area (Libya, Tunisia, Algeria and Morocco) [12]. The prevalence of PDN in people with diabetes has been estimated to be 53.7% in the Middle East region [13], 65.3% in Saudi Arabia [14] and 14% in Turkey [15]. Recently, we conducted a systematic review that included eight surveys of seven countries from the MENA region (Saudi Arabia, Algeria, Egypt, Lebanon, Jordan, Gulf States and Turkey) [16] and estimated the point prevalence of PDN associated with type 1 or type 2 diabetes to be 43.2% (95% CI = 30.1–57.2%, pooled sample = 7898 adults (3761 women), effect size = − 0.949).

In 2017, the IDF predicted that 442,500 people were living with diabetes in Libya (11.2% of the population) [17]. In 2012, Elzahaf et al. [18] published the findings of a systematic review of data from 65 surveys in 34 countries that estimated the prevalence of chronic pain worldwide to be 30.3% ± 11.7%, although they failed to identify any data for Libya. Recently, we conducted a search of research databases and failed to find any epidemiological studies estimating the prevalence of PDN in Libya. Previously we have translated the LANSS pain scale and the self-report version of the Leeds Assessment of Neuropathic Symptoms and Signs (S-LANSS) into Arabic and found both instruments to be valid and reliable to screen for neuropathic pain in a sample of adults with diabetes living in Libya [19]. We estimated the prevalence of PDN to be 41.3%, but expressed caution at this finding because it was derived from a small sample of 109 adult patients. The aim of the present cross-sectional epidemiological study was to estimate the point prevalence of PDN using a larger sample in adults from Eastern Libya and to determine risk factors that contribute to development of the condition.

## Methods

### Study design

This was a cross-sectional study that collected S-LANSS scores and indices of glycaemic control from a sample of patients with diabetes attending the Benghazi Diabetes Centre (BDC), Libya. The BDC has been operational for 48 years and is one of the largest diabetes centres in Libya, servicing the Eastern region of the country. The Centre employs 25 health care professionals, including five physicians dedicated to patient follow-up, with between 300 and 500 consultations each day and a throughput of approximately 100,000 patients per year [20, 21]. Services include reviewing patients’ medication, measuring fasting plasma glucose concentration, checking for complications of diabetes and referral to follow-up clinics for endocrine care, eye care, dental care and diet. There is no follow-up service for painful nor non-painful diabetic neuropathy and limited provision for foot care to prevent gangrene.

Ethical approval to conduct the study was granted from Leeds Beckett University research ethics committee and study-site permission from Benghazi Health Authority. The well-being, privacy, dignity and safety of participants were respected at all times in accordance with the Research Governance Framework and Policy and Practice of Research Ethics of Leeds Beckett University, and Medico-legal principles approved by the Libyan General Medical Council.

### Sample population and sample size

The sample size was calculated using an online calculator (Soft Stat software) based on the minimum sample size needed to estimate the true population proportion with the required margin of error of 0.05% and confidence level at 95%. A sample of 400 participants was required to detect a relationship between the duration of diabetes (independent variable) and S-LANSS score (Arabic version, dependent variable). The level of statistical significance was set at 0.05 (two-sided). A target sample of 450 participants was set to account for attrition due, for example, to withdrawal of participants, with equal numbers of men and women matched for age into five subgroups: 18 < 30 years, 30 < 40 years, 40 < 50 years, 50 < 60 years and ≥ 60 years.

Receptionists and health care team members from the BDC advertised the study verbally. Potential participants were contacted in liaison with the BDC and an information form was distributed. Patients expressing interest in the study were given a participant information pack and were contacted 48 h later to be invited to attend one, one-hour study visit. The process of invitation, selection, inclusion/exclusion and enrolment is described in the study flow chart (Additional file 1).

### Study visit

All eligible volunteers were enrolled onto the study by providing signed consent. Participants were interviewed by the Principal Investigator (SG) and provided data on anthropometrics, socioeconomics and education. If participants answered ‘yes’ to the question “Have you suffered from any pain in your feet in the last 6 months?” they were invited to complete the Arabic version of the S-LANSS pain scale. A fasting sample of venous blood was collected by a trained member of staff from the BDC.

### Data management and analysis

Data was analysed using Statistical Package for Social Science (SPSS) version 22. Data is described as mean and standard deviation unless otherwise stated. Distribution of continuous variables were tested for normality using the kolmogrov-Smirnov statistic. Comparison between two continuous variables (e.g. duration of diabetes of men versus women) were examined using an unpaired t-test if the distribution of the variable was normal, and a Mann-Whitney statistic if the distribution was not normal. Differences between two proportions or two dichotomous variables were tested statistically using Chi-square or Z tests.

Initially, we calculated crude odd ratios of having PDN for groups of participants classified according to; sex, age group, Body Mass Index (BMI), duration of diabetes, fasting plasma glucose concentration and smoking for men only, as all women were non-smokers. The assignment of a reference group in all odds ratio calculations was based on previous literature to allow for meaningful comparisons [22]. We used a logistic regression model to test whether potential risk factors (sex, age, BMI, smoking history for men, duration of diabetes and fasting plasma glucose concentration) predicted probable neuropathic pain (i.e. a total S-LANSS score of ≥ 12). We chose logistic regression because the outcome variable was dichotomous and it would be possible to calculate adjusted odds ratios for all potential predictors. Each independent variable in the model had two categories (e.g. age = ≤ 60 years and > 60 years (old age); BMI = < 30 Kg/m^2^and ≥ 30 Kg/m^2^ (obese); duration of diabetes = 5–15 years and > 15 years; and fasting plasma glucose concentration ≤ 125 mg/dl and > 125 mg/dl). In addition, the association between potential risk factors (i.e. collinearity) was tested using the Spearman correlation coefficient [23].

## Results

### Characteristics of the sample

In total, 549 volunteers were invited to attend a study visit, of which 99 declined for a variety of reasons, often because they were ‘too busy’ or it was ‘too far to travel’. Four hundred and fifty volunteers attended a study visit and all completed the study protocol (age = 19–87 years, BMI = 17.6–44.2 kg/m^2^, 224 women, Table 1). Nine samples of blood were not obtained from participants and recorded as missing data. Women were older than men (mean difference = − 2.79 years, 95% CI = − 5.35-0.23 years), but there was no difference in BMI between women and men (mean difference = 0.55 kg/m^2^, 95% CI = − 0.25-1.33 kg/m^2^). Mean ± SD fasting plasma glucose concentration was 130.35 ± 26.08 mg/dl (*n* = 441 participants) and mean ± SD duration of diabetes was 15.08 ± 7.13 years. There were no differences between women and men in fasting plasma glucose concentration (mean difference = 2.85 mg/dl, 95% CI = − 2.03-7.73 mg/dl) or duration of diabetes (mean difference = 0.64 years, 95% CI = − 0.68-1.97 years). There were 93 smokers (41.2%) and all were men. Fewer women were employed than men.

**Table 1:** Characteristics of participants

Characteristic	Men	Women	Total	*P* value*
Sample size	226	224	450	
Age (years)	49.17 ± 13.99	51.96 ± 13.67	50.56 ± 13.89	0.03^a^
BMI (kg/m^2^)	29.77 ± 3.80	29.23 ± 4.70	29.50 ± 4.28	0.18^a^
Plasma glucose**(mg/dl)	131.77 ± 25.95	128.93 ± 26.18	130.35 ± 26.08	0.17^b^
Duration of Diabetes (years)	15.40 ± 7.08	14.75 ± 7.18	15.08 ± 7.13	0.26^b^
Smoking (*n*, %)	93, 41.2%	0%	20.7%	0.00^c^
Duration of smoking (years)	7.99 ± 11.71	No women smokers	7.99 ± 11.71***	0.00^b^
Employment (*n*, %)	173, 76.5%	126, 56.3%	299, 66.4%	0.00^c^
Education (*n*, %)				
• Cannot read or write	42, 18.6%	50, 22.3%	92, 20%	0.66 ^c^
• Can read and write****	19, 8.4%	36, 16.1%	55, 12.2%	0.43 ^c^
• Primary	30, 13.3%	28, 12.5%	58, 12.9%	0.93 ^c^
• Secondary	40, 17.7%	32, 14.3%	72, 16%	0.70 ^c^
• University or above	95, 42%	78, 34%	173, 38.4%	0.27 ^c^
Marital status (*n*, %)				
• Married (no children)	37, 16.4%	28, 12.5%	65, 14.4%	0.66 ^c^
• Married (children)	139, 61.5%	142, 63.4%	281, 62.4%	0.74 ^c^
• Unmarried	30, 13.3%	27, 21.1%	57, 12.7%	0.44 ^c^
• Others	20, 8.8%	27, 12.1%	47, 10.4%	0.72 ^c^

*Differences between mean ± SD or the proportions of men and women were tested by ^a^ unpaired t-test, if the data was normally distributed, ^b^ Manny Whitney if the data was not normally distributed and ^c^ Chi square to test the differences in between proportions of men and women

**Plasma glucose numbers are 221 for males and 220 for females

***None of the women were smokers, so there is no mean or standard deviation for this group. Values for males only

****can read and write but not formally educated

Two hundred and twenty six participants (50.2%) used insulin on its own for diabetes control. One hundred and sixty three participants (36.2%) used oral hypoglycaemic medications on their own and 61 participants (13.6%) used a combination of insulin and oral hypoglycaemic medication. Fig. 1 presents complications associated with diabetes.

One hundred and ninety five participants answered ‘yes’ to having suffered from pain in the foot in the last six months. Thirty seven of these 195 participants (19%) received over the counter medications (OTCs) without specifying names. Thirty three participants (17%) received paracetamol (PCM), 27 participants (13.8%) received PCM and non-steroidal anti-inflammatory drugs (NSAIDs), 22 participants (11.9%) received tramadol, 22 participants (10.7%) received co-codamol, 19 participants (9.7%) received NSAIDs, 13 participants (6.7%) received codeine, eight participants (4.1%) received herbal medicine, 5 participants (2.6%) received cupping, four participants (2%) received gabapentin and three participants (1.5%) received duloxetine. Three participants reported receiving no treatment at all (1.5%).

### Prevalence of pain with neuropathic characteristics

Of the 450 participants in our sample, 195 (43.3%) reported pain in the previous 6 months. All of these participants reported that this pain was in their lower limbs. Of the 195 participants reporting pain, 190 scored ≥ 12 on S-LANSS. Thus, 42.2% (190/450) of participants with diabetes were categorised as experiencing pain with neuropathic characteristics. Interestingly, only seven of the 195 (3.6%) participants with pain reported that they had been clinically diagnosed with painful diabetic neuropathy.

There was no statistically significant differences in the prevalence of pain with neuropathic characteristics between women and men (odds ratio (OR) = 0.97, 95% CI = 0.67–1.4). Risk factors for the development of PDN were more than 60 years of age (OR = 1.93, 95% CI = 1.22–3.04), longer duration of diabetes (OR = 23.41, 95% CI = 14.26–38.46), had smoked tobacco for more years (OR = 5.60, 95% CI = 3.11–8.60), had higher fasting plasma glucose levels (OR = 3.11, 95% CI = 2.1–4.6), had higher BMI (OR = 4.56, 95% CI = 3.1–6.8) and had higher employment status.

### Multivariate (adjusted) odd ratio

The adjusted odds ratios in the multivariate logistic regression model (Table 2) revealed that the most significant predictors of PDN were duration of diabetes (OR = 25.85, 95% CI = 13.56–49.31), followed by smoking in men (OR = 8.28, 95% CI = 3.53–9.42), obesity (OR = 3.96, 95% CI = 2.25–6.96) and high fasting plasma glucose concentration (OR = 3.51, 95% CI = 1.99–6.21). The effect of sex and age was not significant on the prevalence of PDN (OR sex = 0.24, 95% CI = 0.17–0.48; OR age = 0.54, 95% CI = 0.3–1.1).

**Table 2:** Crude and adjusted odd ratios for the potential risk factors of PDN in Eastern Libya

Predictor binary variables	PDN Present	PDN Absent	Odds ratio (95% CI, *P* value)	Adjusted odds ratio (95% CI, *P* value)
Sex				
Men^a^	96	128	0.97	0.24
Women	95	131	(0.67 to 1.4, 0.86)	(0.17 to 0.48, 0.89)
Age				
≤ 60 years^a^	138	126	1.93	0.54
> 60 years	53	43	(1.22 to 3.04, 0.005)	(0.26 to 1.1, 0.89)
BMI				
< 30 Kg/m^2 a^	66	183	4.56	3.96
≥ 30 Kg/m^2^	125	76	(3.1 to 6.8, < 0.0001)	(2.25 to 6.96, < 0.0001)
Smoking				
Non-smokers^a^	68	25	5.60	8.28
Smokers	123	234	(3.11 to 8.60, < 0.0001)	(3.53 to 19.42, < 0.0001)
Duration of diabetes				
5–15 years^a^	41	224	23.41	25.85
> 15 years	150	35	(14.26 to 38.46, < 0.0001)	(13.56 to 49.31, < 0.0001)
Fasting blood sugar				
≤ 125 mg/dl^a^	65	155	3.11	3.51
> 125 mg/dl	125	96	(2.1 to 4.6, < 0.0001)	(1.99 to 6.21, < 0.0001)

^a^Reference group

## Discussion

This study estimated that 42.2% of individuals with diabetes in Eastern Libya had probable PDN, based on a S-LANSS score of ≥12. This estimate is similar to our pilot study that found that 41.3% of 109 individuals with diabetes had probable PDN [19]. The estimate is similar to the findings of our systematic review that estimated the prevalence of PDN in individuals with diabetes in the MENA region to be 43.2%, (95% CI = 30.1–57.2%, eight surveys, 7806 participants, 3761 women) [16]. Our estimate of prevalence of PDN in Libya is lower than that reported in Saudi Arabia (65.3%) [14] and higher than Turkey (14%) [15] and (23%) [24], but higher than the UK (33%) [25] and the United States of America USA (11–25%) [26].

### Reasons for the differences in estimates between countries

There are variations in the estimates of the prevalence of PDN between and within countries in research literature. Reasons include disparities in study methodologies, sample characteristics, eligibility criteria for co-morbidities, treatments to manage diabetes and operational definitions and measurement techniques to identify neuropathic pain [27–29]. For example, a cross-sectional study conducted in the UK using a postal survey estimated that 64% of individuals with diabetes experienced neuropathic elements but this figure declined to 30% when these respondents were assessed using a neurological examination [27]. There has been a debate about the possibility of imprecise estimates of prevalence due to the use of neuropathic pain screening tools rather than full neurological examination [30–33]. Symptoms-based questionnaires including S-LANSS are widely used to screen for probable neuropathic characteristics by researchers and health care professionals prior to further examination if required. Thus, we decided to use the S-LANSS as it would enable direct comparison with previous studies. We intend to conduct a study using full neurological examination in the future [33].

### Risk factors for the development of PDN

The main risk factors for PDN identified in our study were increased duration of diabetes (> 15 years), obesity (BMI ≥ 30 Kg/m^2^), high fasting plasma glucose concentration (> 125 mg/dl) and smoking (only for men). This is consistent with research findings from other studies in the MENA region [12–15, 24, 34, 35] and worldwide [36]. Other risk factors not measured in our study include hypertension and high cholesterol. Evidence also suggests that there is a higher incidence PDN in individuals with prolonged impaired glucose intolerance before diabetes has been diagnosed [37–39]. Socioeconomic factors including lifestyle, diet, and health care policies such as food labelling and the availability low glycaemic food items facilitating blood sugar level control contribute to higher incidence of diabetes and associated complications and may be one reason for country and regional differences in the prevalence of PDN [29].

Higher levels of education and employment were also associated with higher percentages of people with PDN possibly because they are more likely to have sedentary jobs and higher BMI.

### Clinical implications of the findings

In our study, only 3.6% of individuals with PDN reported that they had received a diagnosis of PDN from their clinician and this was from consultations outside of Libya (Tunisia and Egypt). We speculate that clinicians in Libya may not be aware of the presence of PDN in patients or that they consider PDN to be an inevitable consequence of diabetes and not worthy of specific consideration and/or treatment. Clearly, there is a need to raise awareness of the importance of diagnosing and managing PDN including the consequence of inadequate control of diabetes in future policy. Our findings provide evidence that long-term glycaemic control is critical to reduce the incidence of PDN and that there is inadequate management of pain. This is not unique to Libya. For example, Daousi et al. reported that PDN was inadequately managed in more than 40% of individuals in the United Kingdom [29, 40], with similar findings in Canadian populations [41].

Strong evidence from systematic reviews suggest that PDN can be managed using pharmacological interventions including tricyclic antidepressants and gabapentin or pregabalin as first-line treatments and serotonin-norepinephrine reuptake inhibitors or opioids as second-line treatments [3, 12]. This approach has been endorsed by professional bodies, including the International Association for the Study of Pain (IASP) and the National Institute for health and Care Excellence (NICE) and has been adapted for use in the MENA region [12, 42]. However, treatments received by participants in our sample suggest that this approach was not implemented, with only four participants receiving a first-line drug (gabapentin) and 53 a second-line drug (either tramadol, co-codamol or codeine). Of the remaining participants who received treatment for their pain, some received analgesics not suitable for the neuropathic pain or alternative therapies such as herbal medicine or cupping.

### Strengths and shortcomings of the study

This is the first attempt to estimate the prevalence of PDN in individuals living in Libya using an appropriately powered study with adequate sample size. The absence of diagnosis based on a full neurological examination is a limiting factor, although we estimated that the use of our validated Arabic version of the S-LANSS pain scale would identify at least 75% of individuals with PDN [43]. We measured fasting plasma glucose concentration rather than haemoglobin A1c (HbA1c) which is a more robust indicator of blood sugar control. We did not measure blood pressure, triglycerides and cholesterol levels and such data would have enabled determination of their role as probable risk factors.

The sample may be subject to selection bias because the study site clinic only services the Eastern region of Libya. However, there are no primary services for individuals with diabetes in Libya so individuals have to attend regional specialised clinics. Thus, the sample is likely to have captured all people with diabetes who were willing and able to attend the clinic. Nevertheless, we express caution in the generalisability of our estimate because of the use of a single-site and the impact of unmeasurable confounders.

### Future directions

It is hope that our findings will be used to inform future policy for the diagnosis and management of PDN associated with diabetes in Libya, including the creation of a national prevention program [44]. Future epidemiological research is needed from resource limited countries to provide a more a balanced picture of the problem of PDN globally. There is an increasing focus on epigenetics to identify individuals susceptible to PDN [45], and studies investigating the incidence of microvascular pathology preceding diagnosis of diabetes would provide valuable insights into the pathogenesis of PDN. Microvascular pathology is known to contribute to neuropathy and may have clinical utility as a predictor of PDN.

## Conclusion

The prevalence of PDN in adults with diabetes in Eastern Libya was 42.2% and risk factors for developing PDN were high blood glucose, long duration of diabetes, and higher level of educational and employment status. The prevalence of PDN in Libya is higher than reported in countries in Europe and the USA. We suspect that some clinicians in Libya may consider PDN to be an inevitable consequence of diabetic neuropathy and fail to appreciate the importance of appropriate management.

## Additional file

Additional file 1: A cross-sectional study to estimate the point prevalence of painful diabetic neuropathy in Eastern Libya. (DOCX 37 kb)

## Abbreviations

BDC: Benghazi Diabetes Centre
BMI: Body Mass Index
BPS: British Pain Society
CPS: Canadian Pain Society
DN4: Douleur Neuropathique en 4 questions
EFNS: European Federation of Neurological Sciences (
GBP (£): British Pound Sterling
HbA1c: haemoglobin A1c
IASP: International Association for the Study of Pain
IDF: International Diabetes Federation
LANSS: Leeds assessment of neuropathic symptoms and signs
MENA: Middle East and North Africa
NHS: National Health Service
NICE: National Institute for health and Care Excellence
NPQ: Neuropathic Pain Questionnaire
NSAIDs: Non-Steroidal Anti-Inflammatory Drugs
OR: Odd Ratio
OTC: Over The Counter
PCM: paracetamol
PDN: Painful Diabetic Neuropathy
S-LANSS: Self-report Leeds assessment of neuropathic symptoms and signs
SNRIs: Serotonin-Norepinephrine Reuptake Inhibitors
SPSS: Statistical Package for Social Science
StEP: Standardized Evaluation of Pain
TCAs: Tri-Cyclic Anti-depressants
UK: United Kingdom
USA: United States of America
USD ($): United States Dollar
