# Supplementary material for: A cross-sectional study to estimate the point prevalence of painful diabetic neuropathy in Eastern Libya
Source: BMC Public Health. 2019 Jan 17;19:78. doi: 10.1186/s12889-018-6374-9 (PMC6335782; doi:10.1186/s12889-018-6374-9)
Supplement: Supplementary file 1 — A cross-sectional study to estimate the point prevalence of painful diabetic neuropathy in Eastern Libya. (DOCX 37 kb) [file 12889_2018_6374_MOESM1_ESM.docx]

Advertise study at outpatient diabetic clinic in Benghazi

Initial contact

with patient

Initial Eligibility Check

[Check allocation block free]

Organise study visit

Participant Information Sheet

Organise contact >48h later

Invite (>48h)

Study Visit

Enrolment

Not eligible - exit

Allocation block full - exit

Consent

Study briefing and

Participant Information

Study Visit

Interview

1. Anthropometric data and history of Diabetes

2. Complete S-LANSS pain scale

Exit

Does not wish to take part - exit

Not eligible - exit

Does not wish to take part - exit

Screening Eligibility
